# The Emerging Role of Proteolysis in Mitochondrial Quality Control and the Etiology of Parkinson's Disease

**DOI:** 10.1155/2012/382175

**Published:** 2012-05-13

**Authors:** Riya Shanbhag, Guang Shi, Jarungjit Rujiviphat, G. Angus McQuibban

**Affiliations:** Biochemistry, University of Toronto, 1 King's College Circle, Toronto, ON, Canada M5S 1A8

## Abstract

Mitochondria are highly dynamic organelles that are important for many diverse cellular processes, such as energy metabolism, calcium buffering, and apoptosis. Mitochondrial biology and dysfunction have recently been linked to different types of cancers and neurodegenerative diseases, most notably Parkinson's disease. Thus, a better understanding of the quality control systems that maintain a healthy mitochondrial network can facilitate the development of effective treatments for these diseases. In this perspective, we will discuss recent advances on two mitochondrial quality control pathways: the UPS and mitophagy, highlight how new players may be contributing to regulate these pathways. We believe the proteases involved will be key and novel regulators of mitochondrial quality control, and this knowledge will provide insights into future studies aimed to combat neurodegenerative diseases.

## 1. Introduction: Mitochondrial Quality Control Systems

Parkinson's disease (PD) is a neurodegenerative disease of the central nervous system that results from the loss of dopaminergic neurons in the substantia nigra (SN) region of the brain. While environmental factors such as oxidative and nitrosative stress are responsible for the, more common, sporadic form of PD [1], there are genetic factors that contribute to the familiar (genetic) form of the disease [2]. Both forms of PD are characterized by the formation of Lewy bodies (LBs)—aggregates of various proteins including alpha-synuclein, ubiquitin, and tubulin. Although the mechanism that leads to PD pathogenesis remains elusive, studies have shown that mitochondrial dysfunction plays a role in the progression of both forms of the disease [2–4].

 Mitochondria are vital for many diverse processes in the cell, such as energy metabolism, calcium buffering, and cell apoptosis. These double-membrane-bound organelles are often called the “powerhouses” of the cell because they generate the cell's supply of energy in the form of ATP. They are also the main source of reactive oxygen species (ROS) and therefore are most susceptible to damage by oxidative stress. An accumulation of ROS can lead to mutations in mitochondrial DNA and misfolding and aggregation of proteins, which can disrupt normal functions [5]. It is known that neuronal cells are particularly vulnerable to the functional deterioration of mitochondria, primarily due to their high-energy demands. Consequently, along with PD, mitochondrial dysfunction has been linked to other neurodegenerative diseases such as Alzheimer's and Huntington's disease, and the pathways discussed below may be applicable [6–8].

 Since proper functioning mitochondria are required for various cellular processes, it is not surprising that there are several quality control systems that work to maintain a healthy mitochondrial population. Mitochondria are constantly undergoing fusion and fission events; the exact reason for these dynamics is not currently known, but it is thought they serve to monitor and protect the integrity of the mitochondrial network. Fusion, the merging of distinct mitochondria, enables the mixing and dilution of defective proteins, while fission facilitates the division and the segregation of severely damaged areas [9, 10]. In addition, in each of its compartments, the mitochondrion houses proteases that degrade misfolded and/or dysfunctional proteins [9].

Recent studies have revealed that the ubiquitin proteasome system (UPS) also participates in recycling mitochondrial-associated proteins (see reviews [11–14]). The UPS is a primarily cytosolic system that eliminates impaired and/or nonfunctional proteins in the cell. It involves the covalent attachment of a 76-amino acid protein, called ubiquitin. Ubiquitination of a protein occurs via three sequential steps: (i) ubiquitin activation by E1 enzymes, (ii) conjugation to a carrier by E2 enzymes, and (iii) ligation of ubiquitin to the target by E3 ligases. Once a substrate is tagged with K48-linked polyubiquitin chains, it is degraded by the 26S proteasome. Conversely, deubiquitinating enzymes (DUBs) appear to drive the pathway in reverse by opposing the function of E3 ligases by removing or editing the ubiquitin chains.

While new lines of evidence indicate that the UPS is important for the removal of individual mitochondrial proteins, the autophagy-lysosome pathway (ALP) is responsible for the degradation of the organelle as a whole [15]. Autophagy can be subcategorized into three forms: macroautophagy, microautophagy, and chaperone-mediated autophagy [16]. The form that is of interest to us and the field of mitochondrial dynamics and quality control is a selective type of macroautophagy, called mitophagy, which mediates the removal of damaged mitochondria that can be toxic to the cell [17, 18]. The initial step in mitophagy entails recruiting double-membrane-bound vesicles called autophagosomes [19]. These structures first engulf mitochondria and the surrounding cytosolic constituents and then subsequently fuse with the lysosome, which degrades the sequestered cargo [16]. In yeast, mitophagy can be mediated by the autophagy-related protein Atg 32, amongst several other Atg proteins [20]. In mammalian systems, mitophagy can be initiated through different mechanisms, such as NIX in maturating red blood cells [21] and the PINK1/Parkin pathway [17], which will be discussed in more detail in subsequent sections.

The multiplicity of quality control systems reflects the high level of complexity and intricacy of design, but this makes it difficult to elucidate exactly how mitochondrial dysfunction contributes to neurodegeneration. In this perspective, we will present key discoveries in two distinct yet interconnected mitochondrial quality control systems: the UPS and mitophagy. The hypotheses proposed here, regarding novel regulators within these pathways, will serve as a starting point for further studies on the subject. Notably, we highlight that several important proteases might be implicated in orchestrating the overall pathway of mitochondrial homeostasis. A better understanding of the systems at play and their relationship with mitochondrial metabolism and intrinsic proapoptotic events may lead to the development of novel screening tools and therapeutic treatments for several debilitating neurodegenerative human diseases like PD.

## 2. The UPS and Mitochondrial Protein Degradation

### 2.1. Several Mitochondrial Proteins Are Proteasomal Substrates

The UPS has recently been linked to mitochondrial function as a part of its overall quality control system. The initial evidence that implicates the UPS in mitochondrial protein degradation comes from proteomic screens of mitochondria that have identified over 100 proteins that can undergo ubiquitination [13, 22, 23]. The proteins identified are important for various mitochondrial functions such as ATP production by oxidative phosphorylation and biosynthesis of fatty acids [13]. Protein ubiquitination is a posttranslational modification that can lead to proteasomal degradation, alter specific cellular localization, or control other regulatory outcomes in the cell such as immune signalling and cell cycle and division [24]. In addition, the mitochondrial ubiquitinome may consist of nuclear-encoded proteins that are ubiquitinated for their import into the mitochondria. As shown by Zhaung and McCauley, ubiquitin conjugation of monoamine oxidase B serves as a signal for its import into mitochondria [25].

**Figure 1:**
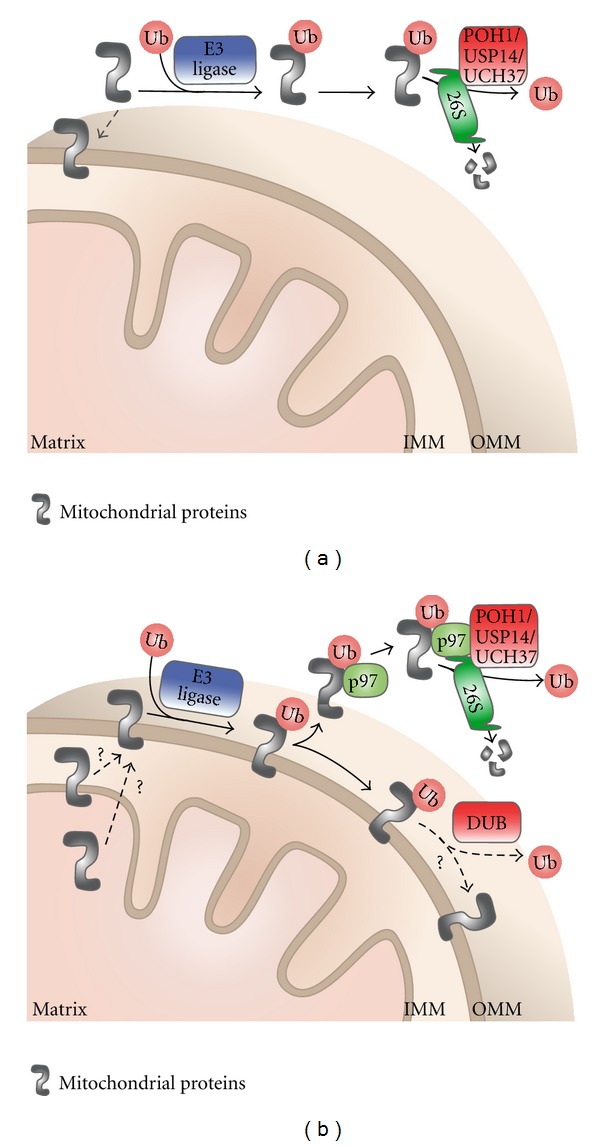
A model of UPS-mediated mitochondrial protein degradation. (a) Damaged and/or misfolded nuclear-encoded proteins that are destined for import into the mitochondria are intercepted by E3 ubiquitin ligases and labeled with K48-linked polyubiquitin chains. Subsequently, these proteins undergo degradation by the cytoplasmic 26S proteasome. Only the substrate alone is degraded, while ubiquitin is recycled by one or more of the proteasomal-specific deubiquitinating enzymes (DUBs, shown in red). (b) The turnover of proteins that are already at the mitochondria occurs via a process called mitochondria-associated degradation (MAD). Defective proteins at the OMM are polyubiquitinated by E3 ligases. These proteins are then extracted from the membrane by the AAA-ATPase p97 and delivered to the proteasome for proteolysis. Alternatively, mitochondrial-associated DUBs may rescue proteins from degradation by editing or removing the degradative ubiquitin signal. Hence, DUBs may enhance the stability of mitochondrial proteins. In addition, many lines of evidence suggest that the UPS also facilitates the degradation of non-OMM proteins. However, the mechanistic details of how proteasomal substrates within inner compartments retrotranslocate to the OMM have not yet been established.

The mitochondrial ubiquitinome may also represent proteins that have been marked for clearance by the cytoplasmic 26S proteasome. There is increasing evidence to support this latter view that the proteasome functions to eliminate defective proteins in the cytosol that were destined for the mitochondria to prevent the import of damaged cargo (Figure 1(a)). Alternatively, and perhaps simultaneously, the proteasome is responsible for the degradation of proteins at the mitochondria, more specifically proteins on the OMM that share an interface with the cytoplasm. Well-studied examples of OMM proteins that are degraded in a proteasome-dependent manner include Mcl1, a prosurvival member of the Bcl-2 family [26, 27]; Drp1, a mitochondrial fission protein [28]; mitofusins 1/2, regulators of mitochondrial fusion [29, 30]. These observations highlight the influence of the UPS in regulating apoptosis and mitochondrial membrane dynamics. It is likely that these processes will also be required for several homeostatic pathways during normal development and cellular differentiation, underscoring the importance of proteolytic regulators.

### 2.2. Non-OMM Proteasomal Substrates

Surprisingly, new research has shown that the UPS may also play a role in the degradation of non-OMM proteins. Margineantu et al. reported that proteasome inhibition with MG132 leads to increased mitochondrial mass, which may be attributed to an accumulation of IMM-localized proteins [31]. Consistent with these findings, specific targets of the proteasome that have been identified include endonuclease G, an IMS protein [32]. Additionally, two integral IMM proteins, UCP2 and 3, were shown to be degraded by the 26S proteasome after undergoing K48-linked polyubiquitylation [33, 34]. Remarkably, it was found that the mitochondrial matrix localized oligomycin sensitivity-conferring protein (OSCP) is also degraded in a proteasome-dependent manner, with the help of hsp90 [31]. Taken together, these studies indicate, contrary to popular belief, that proteasome-mediated degradation is not restricted to OMM proteins; proteins in the IMS, IMM, and matrix are also candidate substrates for the 26S proteasome (Figure 1(b)).

### 2.3. Molecular Steps of the UPS at Mitochondria

 The exact mechanisms that govern proteasome-mediated turnover of mitochondrial proteins remain undefined. Nevertheless, there is a proposed pathway that is based on another membrane-enclosed organelle, the endoplasmic reticulum (ER). The ER-associated degradation (ERAD) pathway is a multistep process that allows the 26S proteasome to extract and breakdown proteins that are normally confined by the ER membrane. This process requires Cdc48 (known as p97 in mammals) and the adaptor proteins Np14 and Ufd1 [11]. Similarly, following a loss of mitochondrial membrane potential, p97 has been shown to accumulate on the mitochondria in order to chaperone polyubiquitinated proteins to the proteasome, in a process called mitochondria-associated degradation (MAD) (Figure 1(b)) [30, 35]. In yeast, under stress conditions, Vms1 is translocated from the cytosol to the mitochondria, where it engages Cdc48/p97 [36]. Although mammalian Vms1 has been shown to purify with p97, it is unclear whether it is required to recruit p97 and initiate MAD. Interestingly, a subset of p97 localizes to the mitochondria under normal conditions, suggesting that it is required to maintain a steady state of proteasomal degradation likely promoting a homeostatic checkpoint to ensure mitochondrial fidelity.

Several questions remain to be addressed regarding the molecular pathway of the mitochondrial UPS. The molecular machinery that retrotranslocates proteins from the inner compartments to the OMM has not been defined. Like with ERAD, perhaps there are additional proteins or cofactors that complete the p97 complex at the mitochondria that have yet to be discovered. In addition, it is also possible that different factors contribute to regulate MAD under normal versus stress conditions. Although we do not yet have a clear understanding of the mitochondrial UPS, the studies to date suggest that multiple independent pathways work in parallel to facilitate the protease-mediated degradation of impaired proteins that reside in, or are *en route* to, the mitochondria.

### 2.4. Components of the UPS at Mitochondria

The hypothesized pathway to regulate mitochondrial health can be further understood by examining individual components of the UPS system, in particular, the E3 ubiquitin ligases. The human genome encodes for ~617 putative E3 ubiquitin ligases [37]. These E3s are classified into several families, with RING finger and BTB proteins being the most abundant in mammalian systems. Since we know that ERAD is driven by ER membrane spanning RING domain E3 ligases, the presence of similar E3s on the OMM would lend support to the MAD pathway. Indeed, a subcellular localization screen of 54 human RING-containing E3 ligases found nine that localized to the mitochondria [38]. Two of these nine, MITOL and MULAN, have been well documented in regulating mitochondrial activity. As shown by Yonashiro et al., MITOL ubiquitinates two mitochondrial fission factors Drp1 and Fis1 [39]. A study on MULAN, an NF-kappaB activator, revealed that it regulates mitochondrial dynamics and signalling [37]. While RING finger E3 ligases are important for ERAD, they might not be a requirement for ubiquitination at the mitochondria. As a case-in-point Mule is an HECT domain containing E3 ligase that ubiquitinates the OMM protein Mcl1 at 5 lysines [40]. Clearly mitochondrial ubiquitin dynamics will be crucial in regulating aspects of mitochondrial biology and cellular metabolism.

### 2.5. Deubiquitinating Enzymes: Crucial Players in the UPS

Ubiquitin conjugation can be reversed by a conserved family of deubiquitinating enzymes (DUBs). Currently, 79 DUBs have been identified in the human genome that are predicted to be effective in counteracting the activity of E3 ubiquitin ligases [41]. These DUBs have been classified into five families, primarily based on structure. They are: the ubiquitin-specific proteases (USPs), the ubiquitin C-terminal hydrolases (UCHs), the ovarian tumour proteases (OTUs), the Machado-Joseph disease protein domain proteases (MJDs), and the JAMM motif proteases (JAMMs) [42].

A wide range of functions have been ascribed to these deubiquitinases, including (i) generating free ubiquitin from ubiquitin precursors, (ii) rescuing proteins from degradation, (iii) editing the ubiquitin biochemical signal, and (iv) recycling ubiquitin from proteasomal substrates [43]. These specific functions enable DUBs to participate in several cellular pathways, including proteolysis, kinase signalling, cell cycle, DNA repair and endocytosis [44]. For example, the three proteasomal DUBs, POH1, USP14 and UCH37, regulate chain lengths, and remove or process ubiquitin so that it is not degraded along with the substrate [45]. Hence, these proteases can rescue proteins and also maintain a steady level of ubiquitin in the cell (Figures 1(a) and 1(b)). Two DUBs, A20 and CYLD, have been well characterized with respect to the NF-kappaB signal transduction pathway, which is required for inflammation and immune responses [46]. A20 and CYLD deubiquitinate K63-linked polyubiquitinated proteins and consequently downregulate signalling [47, 48]. Several DUBs, such as BAP1 and USP44, have been linked to cell cycle regulation [49, 50], while USP1, USP11, and USP28 are important for DNA repair [51–53]. The regulatory role of DUBs in diverse cellular processes has implicated them in many types of cancer and neurological diseases [54].

There are several layers of specificity that enable DUBs to carry out their biochemical activities. DUBs have been shown to exercise specificity based on ubiquitin linkage types and also protein substrates. The above-mentioned USP14, for example, selectively cleaves K48-linked polyubiquitin chains [55]. USP14 activity is important for adequate monoubiquitin levels at pre- and postsynaptic terminals [56]. Moreover, a mutation in USP14, associated with ataxia, has been shown to cause defects in synaptic transmission that lead to paralysis and early death in mice [57]. On the other hand, BAP1 is an example of a substrate-specific DUB that regulates cell proliferation by directly binding to host cell factor-1 and removing its ubiquitin chains [58]. In addition, DUBs can selectively hydrolyse ubiquitin from the ends (*exo*), from within (*endo*), or from monoubiquitinated substrates [43]. This versatility in substrate recognition reflects the means by which DUBs can perform more than one function in multiple cellular pathways. The complexity and elegance of this reversible modification underscore the potential roles for DUBs in regulating important cellular outputs.

### 2.6. Mitochondrial-Associated Deubiquitinases

At present, four mitochondrial-associated DUBs have been identified. The first of these to be identified was USP30, a member of the USP family that resides in the OMM. Nakamura and Hirose reported that USP30 is an integral membrane protein with an N-terminal transmembrane (TM) domain and C-terminal catalytic domain that faces the cytosol [59]. This orientation provides access to substrates on the OMM and the cytosol. Their study also showed that the downregulation of USP30 by RNAi leads to elongated and interconnected mitochondria; thus, it can be deduced that the DUB plays a direct or indirect role in regulating mitochondrial dynamics. Although specific substrates have not been identified, USP30 can cleave both K48- and K63-linked ubiquitin chains [60]. Furthermore, it has recently been shown to interact with p97 [35], which suggests that it may participate in the MAD pathway. Taken together, these observations implicate USP30 in more than one mitochondrial directed activity.

In addition to USP30, USP36 is another DUB that localizes to the mitochondria, albeit not exclusively. It is primarily localized to the nucleoli, where it regulates nucleolar activity through several protein substrates, such as nucleophosmin/B23 and fibrillarin [61]. Recently, it was found that USP36 also localizes at the mitochondria and that it has a strong affinity for mitochondrial manganese superoxide dismutase (SOD2) [62]. More specifically, the overexpression of USP36 decreases the level of polyubiquitinated SOD2 and as a result stabilizes and extends the half-life of SOD2. These two examples clearly implicate the DUB proteases in the regulation of both the activity and the health of the mitochondrion.

Another member of the same family, USP9x, also partially localizes to the mitochondria. It enhances the stability of Mcl1, the previously described antiapoptotic OMM protein, by cleaving K48 polyubiquitin chains [63]. USP9x is the human ortholog of the *Drosophila* DUB, Faf, which was the first DUB linked to cell differentiation in the nervous system [64]. Like Faf, USP9x is thought of be involved in neuronal fate determination and synaptic function through its interaction with epsin-1 [65]. Interestingly, USP9x protein expression was altered in a 1-methyl-4-phenyl-1,2,3,6-tetrahydropyridine-(MPTP-) induced PD mouse model study that measured protein abundance changes, suggesting that it might be associated with neurodegeneration [66]. While these are compelling indications that these important proteases play key roles in mitochondrial function, more work is needed to establish the role of DUBs at the mitochondria and in the nervous system.

Finally, a small pool of the Machado-Joseph disease-associated protein, ataxin-3 also localizes at the mitochondria [67]. Ataxin-3 has the ability to cleave multiple ubiquitin chain types, including K6, K27, K29, K48, and K63, although it has a preference for K63-linked polyubiquitin [68, 69]. It interacts with Parkin, a Parkinson's disease-linked E3 ligase, to counteract several types of ubiquitin conjugations. Hence, it is likely that ataxin-3 edits ubiquitin chains to target Parkin to different cellular pathways such as DNA repair and autophagy (see Section 3). In a *Drosophila* study, ataxin-3 exhibited neuroprotective properties by suppressing polyglutamine-related neurodegeneration [70]. It has not yet been determined whether ataxin-3 possesses similar characteristics within mammalian systems, but again these data provide exciting evidence for DUB activity in regulating neuronal outputs.

**Figure 2:**
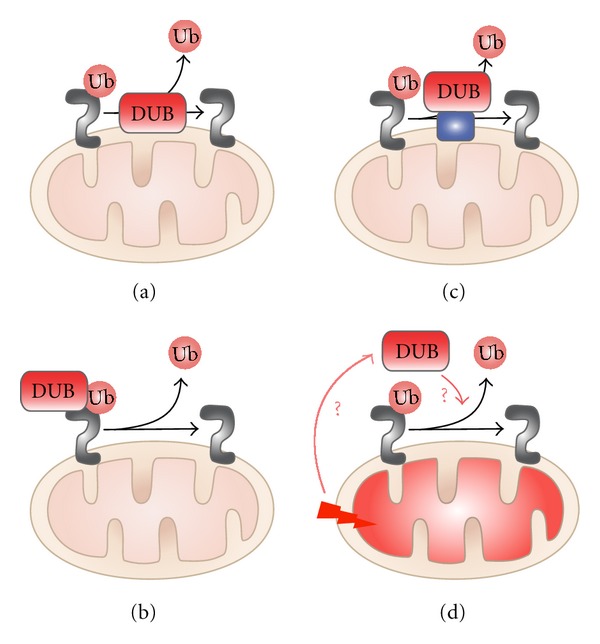
Potential mechanisms to direct deubiquitination at the mitochondria. The diagram illustrates the multiple ways in which DUBs may function at the mitochondria. (a) DUBs such as USP30 can reside in the OMM, in an orientation that provides access to ubiquitin-modified OMM proteins. (b) Substrate-specific DUBs, like USP9x and ataxin-3, might be directly recruited to the mitochondria by their substrates, Mcl1 and Parkin, respectively. (c) It is also possible that DUBs are affixed at the membrane through protein interactions with nonsubstrate proteins at the OMM. This arrangement can bring deubiquitinases in close proximity to their substrates. Although DUBs that use such a mechanism have not yet been identified, the finding that most DUBs contain multiple protein-protein interaction domains lends support to this view. (d) Certain cellular conditions, such as ROS-induced stress, may recruit cytoplasmic DUBs to the mitochondria to counteract the damage.

Deubiquitinases may function at the mitochondria via multiple mechanisms. Like mitochondrial E3 ligases, DUBs might reside in the OMM (Figure 2(a)). But this is only probable for DUBs that contain a TM domain. At present, there are only two with predicted TM domains; interestingly one of them is USP30 (the other is USP19). Alternatively, DUBs may be directly targeted to the mitochondria by their OMM substrates (Figure 2(b)). Three of the above-discussed deubiquitinases, USP36, USP9x, and ataxin-3, likely belong in this category as they appear to be substrate specific. However, considering that there are relatively few DUBs available to oppose the function of over 600 E3 ligases, it is unlikely that all DUBs are substrate specific. A third possibility may exist whereby deubiquitinases are anchored to the mitochondria through interactions with nonsubstrate proteins. Individual proteins or a complex at the OMM could stabilize a DUB, bringing it in close proximity to its substrates (Figure 2(c)). In support of this model, the domain architecture of most DUBs reveals the presence of multiple domains that could mediate such protein-protein interactions [43]. Finally, certain cellular conditions, such as ROS-induced stress, may act as signals to recruit cytoplasmic DUBs to the mitochondria (Figure 2(d)). To summarize, the data on mitochondrial-associated DUBs implicates them in a varied array of mitochondrial and cellular activities.

### 2.7. Future Perspectives on DUBs and Mitochondrial Function in Health and Disease

As advances in the field of mitochondrial protein quality control unravel the roles of the UPS at the mitochondria, another fascinating area that deserves more investigation is the process of deubiquitination. For known mitochondrial deubiquitinases, like USP30, biochemical and cell-based techniques should be used to identify the substrates to understand the downstream physiological significance and the pathways that are regulated by these proteases. Furthermore, DUB genomic and proteomic studies can be used to discover additional mitochondrial-associated DUBs. One such comparative proteomic study identified 6 DUBs that are predicted to localize to the mitochondria (Table 1); none of which have been confirmed [41]. Future studies should be aimed at validating these findings.

In addition to identifying and characterizing individual players: mitochondrial DUBs and their substrates, it is important to ascertain the pathways that these players are involved in. There are several yet uncharacterized stages within mitochondrial pathways where DUBs can intersect (Figure 5). For instance, DUBs may monitor the activity of mitochondrial E3s and hence regulate protein quality control by the proteasome. It is also possible that SUMO-specific DUBs act at the mitochondria in order to regulate mitochondrial fission, while other DUBs may influence mitochondrial fusion factors, and thus, balance membrane dynamics events. With these proposed roles, it is evident that the scope and relevance of DUBs as regulators of ubiquitin-mediated pathways is only beginning to emerge. In the even bigger picture, we note that DUBs are involved in several central mitochondrial metabolic pathways (Figure 6). Metabolism is currently undergoing a renaissance based on the fact that several developmental programs (like cellular differentiation) and several human diseases (like cancer) are orchestrated by fundamental changes in the metabolome. The mitochondrion, and the functions highlighted in Figure 6, likely serves as a central platform to integrate signalling pathways that impact diverse cell biological outputs. It is hoped that further study on mitochondrial DUBs will enhance our current understanding of mitochondrial dysfunction.

## 3. Mitophagy

### 3.1. The Role of Proteolysis in Regulating Mitophagy

Mitochondria are the major sites of ROS production in the cell, and this working-place hazard has created the need for several quality control systems. The most recently identified and characterized pathway is the PINK1/Parkin pathway. This pathway has received much attention as the key molecules are well-known PD-linked genes, and this has directly implicated dysregulated mitochondrial function as a potential cause and therefore therapeutic target for the treatment of PD.

### 3.2. PINK1, Parkin, and Mitophagy

As mitophagy is a mitochondrial quality control system, its failure is thought to trigger the degeneration of neurons, which is considered a hallmark of PD. Although most cases of PD are sporadic, about 10% of cases are genetically inherited [78]. Detailed genetic studies on these cases have provided further evidence to strengthen the relationship between mitophagy and PD. Specifically, researchers have identified that the products of the PD-linked genes, *Park 2* and *Park 6,* are involved in the mitophagy pathway. *Park 2* encodes Parkin, an E3 ubiquitin ligase, which as previously mentioned is an important component of the UPS [79, 80]. *Park 6* encodes a mitochondrial kinase PTEN-induced kinase 1 (PINK1) that plays a role in regulating stress-induced apoptosis and mitochondrial morphology [81–85]. These two proteins have been proposed to function together to mediate the removal of mitochondria by mitophagy as detailed below [86–88]. Studies on both PINK1 mutant *Drosophila* and mouse brains revealed specific functional defects in complex I activity [89]; the same defect in complex I activity has also been found in Parkin-mutant zebrafish [90]. Human neurons lacking PINK1 demonstrate features of marked oxidative stress with widespread mitochondrial dysfunction and abnormal mitochondrial morphology [91]. Interestingly, deficiency of mitochondrial complex I and excessive oxidative damage are frequently observed in neurons of sporadic PD patients [92]. In fact, impairment of mitochondrial quality control, such as mitophagy, has been recently proposed to be one of the mechanisms that trigger the neurodegenerative process [4]. Together, these findings suggest that common mechanisms may underlie both familial and sporadic forms of PD, and impaired mitochondrial clearance could be one such mechanism. Thus, understanding how PINK1 and Parkin mediate and regulate mitophagy may provide valuable insight into PD pathogenesis in both forms of the disease.

### 3.3. Parkin Translocates to Damaged Mitochondria to Initiate Mitophagy

 Under normal conditions, Parkin localizes in the cytoplasm. However, upon the induction of mitochondrial depolarization, Parkin translocates from the cytoplasm to the mitochondria [93]. This translocation has been shown to be very selective, occurring only for damaged mitochondria, but not healthy ones [94]. Since we know that Parkin accumulates on damaged mitochondria and is required for their clearance, it is proposed that Parkin mediates a quality control pathway in order to maintain the fidelity of the mitochondrial network [17].

**Figure 3:**
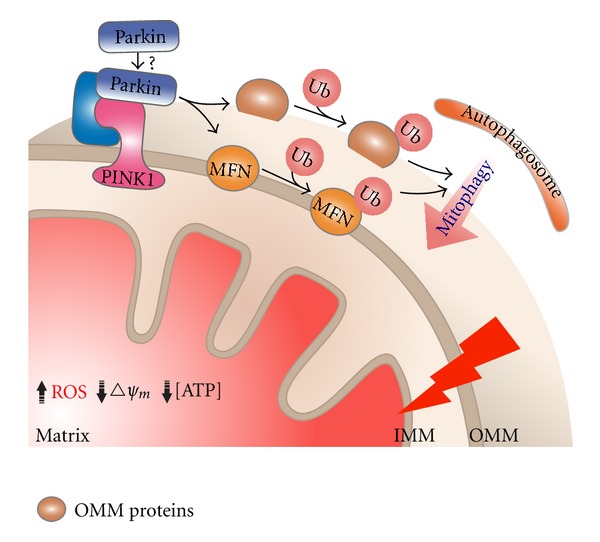
The PINK1/Parkin pathway mediates mitochondrial elimination via mitophagy. Accumulation of ROS and other toxic molecules can damage mitochondria, resulting in the loss of mitochondrial membrane potential and eventually the reduction in mitochondrial ATP concentration. Loss of mitochondrial membrane potential triggers the accumulation of PTEN-induced kinase 1 (PINK1) on the mitochondrial outer membrane (OMM). The accumulation of PINK1 recruits the cytoplasmic E3 ubiquitin ligase, Parkin, to the damaged mitochondria. The mechanism of this recruitment is still unclear; however, several models have been proposed, in which the substrates shared by PINK1 and Parkin may play a role in mediating their interaction. Once on the mitochondria, Parkin ubiquitinates various OMM proteins, including the mitochondrial profusion protein, mitofusin 1. Polyubiquitin chains on these proteins serve as a signal for the recruitment of the autophagosome that engulfs and degrades the damaged mitochondrion through a process called mitophagy.

When recruited to damaged mitochondria, Parkin mediates the ubiquitination of several of its substrates located on the OMM [12] (Figure 3). To date, 5 Parkin mitochondrial substrates have been identified: mitofusins 1 and 2, voltage-dependent anion channel, fission 1, and Tom 20 [29, 95–97]. By ubiquitinating these substrates, Parkin has been proposed to mediate mitophagy using two mechanisms. First, ubiquitination of these OMM localized proteins serves as a signal to recruit autophagosomes to the damaged mitochondria [12]. In the second mechanism, Parkin-dependent ubiquitination could lead to the degradation of its substrates via the UPS [12]. Degradation of its profusion substrate, mitofusin 1, for instance, will lead to mitochondrial fragmentation, making mitochondria more susceptible to mitophagy [29, 95, 98]. Taken together, these findings suggest that Parkin could be a link between the UPS and the ALP, two distinct protein degradation systems in cells. Interestingly, beside its function in the mitochondrial quality control pathway, Parkin has also been proposed to play a role in maintaining normal mitochondrial energy metabolism. In mouse models, gene deletion of Parkin results in a reduction in mitochondrial respiration and an increase in oxidative damage [99]. Future studies on the roles of Parkin in both pathways will help to elucidate the connection between these pathways as well as their roles in cellular metabolism.

### 3.4. Parkin Is Recruited by PINK1 to Damaged Mitochondria

 Studies in fly models have revealed that PINK1 and Parkin function in the same genetic pathway [100]. This interaction has been extended to mammalian systems, as several independent groups have shown that PINK1's activity is required for the translocation of Parkin [101–103]. Interestingly, mitochondrial accumulation of PINK1 or just overexpression of PINK1 can result in the translocation of Parkin and thus mitophagy, even in the absence of mitochondrial uncouplers [88, 94, 104]. In the current model (as illustrated in Figure 3), upon mitochondrial depolarization, PINK1 recruits Parkin to damaged mitochondria and induces their removal by mitophagy [17, 105]. In addition to cooperatively functioning with Parkin in the mitophagy pathway, PINK1 has been proposed to also play important roles in mitochondrial energy metabolism. PINK1 deficiency or PD-linked mutations impair the function of the mitochondrial respiratory chain complex I, resulting in elevated oxidative damage and increased sensitivity to apoptotic stress in mammalian cells and tissues [106, 107]. Recently, using a *Drosophila* model, Liu et al. proposed that PINK1 regulates the oxidative phosphorylation machinery via mitochondrial fission [108]. By participating in multiple pathways, PINK1 could serve as an important link between mitophagy, apoptosis, and cellular metabolism.

Even though there are a large number of studies on the PINK1/Parkin pathway that have been published recently, the fundamental question of how PINK1 recruits Parkin remains unclear. Studies on cellular processing and localization of PINK1 could lay a path to a more clear understanding of PINK1/Parkin-mediated mitophagy. In addition, PINK1 selectively accumulates on damaged mitochondria to recruit Parkin; however, little is known about how this selectivity is achieved. Recent studies on the proteolysis of PINK1 have provided some insights into this selective accumulation of PINK1.

### 3.5. PARL Is a New Player in the PINK1/Parkin Pathway

 PINK1 undergoes proteolysis and rapid proteasomal degradation in many cell types and, as a result, exists at low levels in a healthy cell [109, 110]. However, upon the loss of mitochondrial membrane potential, large amounts of PINK1 can be detected on the mitochondria [94, 103]. It has been proposed that the inhibition of the proteolysis of PINK1 could be a mechanism for selective PINK1 mitochondrial accumulation [81, 89]. Using mouse models, we and other groups have shown that PINK1 undergoes a presenilin-associated rhomboid-like (PARL) dependent proteolysis [93, 111–113]. This proteolysis is highly conserved; we had previously demonstrated that in Drosophila PINK1 is cleaved by rhomboid-7, which is the PARL homolog [100]. We further placed the fly PARL in the same genetic pathway as PINK1/Parkin, strongly implicating this protease in regulating mitophagy. PARL is not the only protease that cleaves PINK1 [112–114]; however, under normal conditions, only the PARL-cleaved PINK1 form is found in the cytoplasm, where it undergoes rapid proteasomal-dependent degradation [111]. In addition, in PARL−/− cells, upon CCCP-induced mitochondrial depolarization, Parkin recruitment and mitophagy are both impaired [111]. Taken together, these data strongly suggest that PARL could be a regulatory member of the PINK1/Parkin mitophagy pathway.

 PARL dysfunction has been previously linked to PD, through its roles in maintaining normal mitochondrial function, regulating apoptosis and mitochondrial morphology [115, 116], and now this linkage is strengthened by its role in mitophagy. Hence, understanding how PARL is regulated may provide us with some valuable insight into the mechanism of PD.

 The regulation of PARL is not fully understood, but PARL undergoes a self-regulated proteolysis at the N-terminus, known as *β*-cleavage. This cleavage event is known to regulate PARL's activity [117, 118]. Interestingly, this cleavage is regulated by phosphorylation of PARL's N-terminus, and the amino acid residue serine (77) is required for this cleavage [117]. We have recently identified a PD-linked mutation that results in a substitution of this serine to asparagine, which abolishes *β*-cleavage [111]. Interestingly, expression of this mutant PARL cannot rescue the Parkin recruitment defect found in PARL−/− cells [111]. Together, these findings suggest a potential role of *β*-cleavage in the PINK1/Parkin pathway. Thus, how *β*-cleavage regulates PARL's activity could be an interesting area to study to further understand mitophagy as a mechanism of disease in PD.

### 3.6. How Does Proteolysis Regulate Mitophagy?

**Figure 4:**
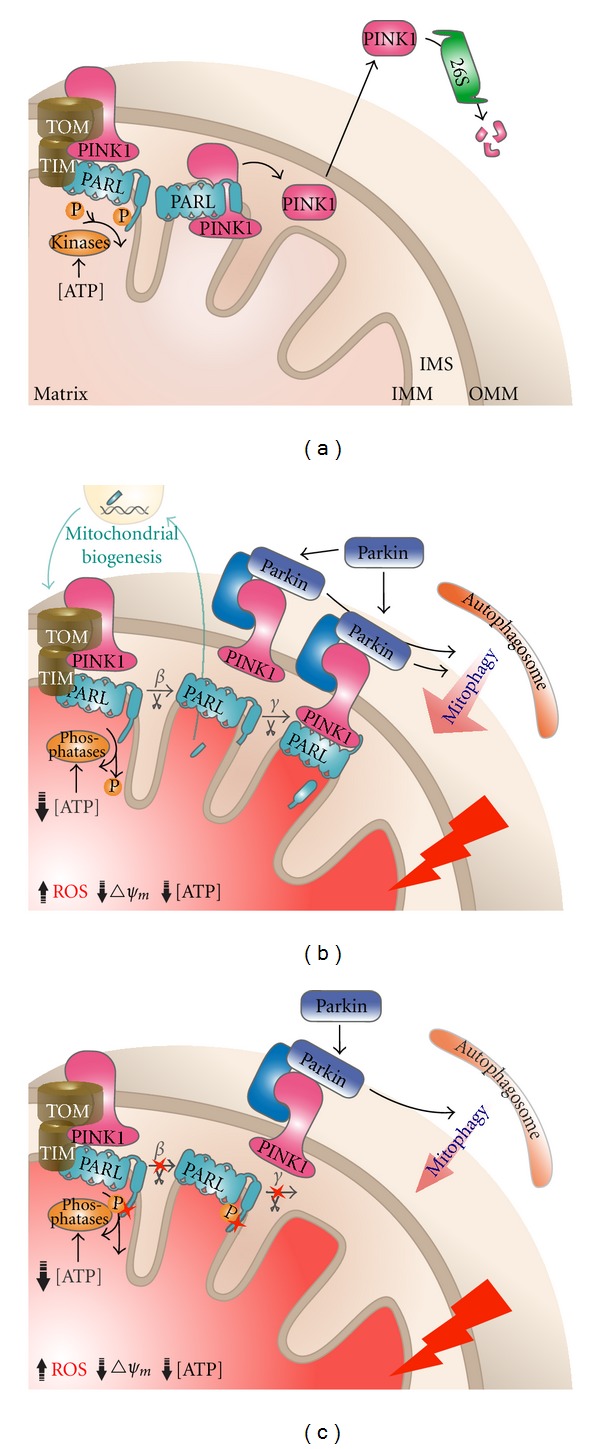
Proposed model for how PARL regulates PINK1 localization under normal conditions and during stress. (a) In a healthy mitochondrion, newly synthesized PINK1 is imported by the TOM/TIM complex. Once it is tethered to the inner mitochondrial membrane (IMM), PINK1 is effectively cleaved by the mitochondrial rhomboid protease PARL, which associates with the TOM/TIM protein-importing machinery. This PARL-dependent cleavage releases PINK1 into the intramembrane space (IMS). The cleaved PINK1 is then retrotranslocated to the cytoplasm, where it undergoes rapid proteasomal degradation. Normal mitochondrial ATP levels facilitate the phosphorylation of the PARL N-terminus. These phosphorylations inhibit the self-regulated proteolysis (*β*-cleavage) that releases the PARL N-terminal domain. (b) During stress, such as accumulation of ROS and the depolarization of mitochondrial membrane potential, mitochondrial ATP levels drop significantly as a result of an inhibition of the electron transport chain. Reduced ATP levels result in the dephosphorylation of the PARL N-terminus, which leads to *β*-cleavage. *β*-cleavage generates a short nuclear-targeted peptide, p*β*, which activates the expression of several genes involved in cell metabolism and mitochondrial biogenesis. Another product of *β*-cleavage, the N-terminal cleaved PARL, then undergoes another cleavage at the loop connecting its first and second transmembrane domains. This cleavage, known as *γ*-cleavage, separates the first transmembrane domain from the rest of the protein and leaves a catalytically dead PARL. Disruption of membrane potential also inhibits the import of PINK1; instead of being imported to the IMM, PINK1 stalls at the import machinery. Because of its association with the TOM/TIM complex, the catalytically dead PARL stabilizes PINK1 on the OMM to recruit Parkin. Parkin triggers the recruitment of autophagosomes, which eliminates the damaged mitochondria through a process called mitophagy. Through other mechanisms, PINK1 may be anchored on the OMM but with a lower efficiency. (c) When *β*-cleavage is inhibited, PARL retains its uncleaved form even when the mitochondrial ATP levels are low. Therefore, during stress, PARL with impaired *β*-cleavage can no longer facilitate the OMM-anchoring of PINK1. Although PINK1 may be anchored to the OMM by other mechanisms, due to their low anchoring efficiency, there will be less OMM anchored PINK1. As a result, Parkin recruitment to damaged mitochondria as well as subsequent mitophagy will be impaired. As a mitochondrial quality control system, failure of proper mitophagy would lead to an accumulation of damaged mitochondria and eventually trigger the cell death pathway, which is one of the contributors to neurodegeneration in PD.

**Figure 5:**
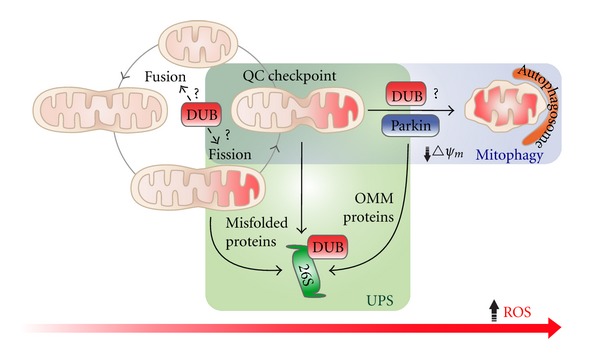
Mitochondrial quality control systems. Mitochondria are dynamic organelles that continually undergo membrane fission and fusion events to maintain their integrity. Under mild to moderate stress, fission facilitates the segregation of damaged areas of the mitochondrial network, while fusion dilutes damaged elements by combining mitochondrial pools. When the damage is extensive, mitochondria are removed by mitophagy. In addition, there is increasing evidence indicating that the UPS participates in the turnover of several OMM and non-OMM proteins. Ubiquitin has been shown to be a key regulator of membrane dynamics and mitophagy. Hence, it is likely that DUBs also participate at various stages within these mitochondrial quality control pathways, in order to maintain a steady equilibrium of protein ubiquitination.

Since *β*-cleavage is a vertebrate-specific process, it may carry some unique functions in higher organisms [117]. It has been shown that *β*-cleavage is required for mitochondrial fragmentation induced by PARL [118]. Since mitochondrial fragmentation promotes mitophagy [30, 119], we propose that the *β*-cleavage of PARL may regulate mitophagy (Figure 4). A product of *β*-cleavage, a short nuclear-targeted peptide p*β*, has been shown to increase the level of PARL and proteins that are involved in mitochondrial biogenesis [120], suggesting that *β*-cleavage could be a mechanism that cells adopt to overcome mitochondrial damage. Upon mitochondrial damage, *β*-cleavage may promote the elimination of damaged mitochondria by isolating them from the rest of the mitochondrial network by fragmentation. In parallel, *β*-cleavage also generates p*β* that upregulates the expression of genes responsible for mitochondrial biogenesis (Figure 4(b)). To test this hypothesis, future studies should focus on elucidating how *β*-cleavage regulates PARL's activity and its downstream effects on mitochondrial morphology and mitophagy.

 Following *β*-cleavage, PARL has been proposed to undergo another cleavage called *γ*-cleavage [121]. Γ-cleavage occurs in a *β*-cleavage-dependent manner; the cleavage generates what is believed to be a catalytically dead rhomboid domain and PARL's first transmembrane domain, TMA [121]. The functional significance of *γ*-cleavage has not been well established. Given the potential role of *β*-cleavage in mitophagy [111], we hypothesize that *γ*-cleavage could also play a role in mitophagy by influencing the selective PINK1 accumulation on mitochondria, one possible mechanism is that, upon mitochondria damage, PINK1 is anchored and stabilized by the catalytically dead rhomboid domain (Figure 4(b)). Thus, it would be interesting to study the functions of the rhomboid domain and TMA, in the context of mitophagy.

 The yeast PARL ortholog, Rbd1/Pcp1, catalyzes an ATP-dependent proteolysis of Mgm1 that releases its soluble form into the intermembrane space [122–124]. In mammals, it is still unclear whether PARL cleaves its substrate in a similar manner. However, there is some evidence suggesting that PARL is involved in regulating cellular metabolism. Downregulation of PARL in human muscle cells results in reduced oxygen consumption, increased oxidative damage, and impaired insulin signalling, suggesting PARL could be a factor in determining oxidative capacity [120]. As mentioned earlier, *β*-cleavage is regulated by the phosphorylation of three amino acid residues at the PARL N-terminus [118]. In mitochondria, the only known kinase/phosphatase pairs are the pyruvate dehydrogenase kinase/phosphatase and the branched-chain keto-acid dehydrogenase kinase/phosphatase, which are involved in regulating proteins in energy production [125, 126]. Thus, future studies should focus on identifying kinase/phosphatase pairs that regulate *β*-cleavage. This will lead to a better understanding of the regulation of PARL and perhaps also reveal new functions for it in other cellular processes. If the regulation of *β*-cleavage is indeed linked to the mitochondrial energy state, we would hypothesize that PARL could sense changes in the mitochondrial energy state, such as ATP levels, and trigger *β*-cleavage and sequential *γ*-cleavage, in order to ensure the well-being of the mitochondrial population via mitophagy and further regulate mitochondrial biogenesis.

## 4. Quality Control Failure and Neurodegeneration

Failures of the UPS are likely linked to PD neurodegeneration in multiple ways. The UPS has been directly linked to sporadic forms of PD, where UPS failure is associated with an accumulation of alpha-synuclein, a hallmark of the disease [127]. It has been established that the proteasome is responsible for the degradation of alpha-synuclein [128]; proteasomal inhibition leads to synuclein inclusions in the cytoplasm [129]. Furthermore, SN dopaminergic neurons of PD patients show reduced expression of the 20S proteasomal alpha-subunit, in comparison to age-matched controls [130]. Although the alpha-subunit is not responsible for enzymatic activity, it is required for proteasomal stability and function. In addition, it is also possible that UPS impairment indirectly elicits neuronal cell death and triggers PD, by contributing to mitochondrial dysfunction. Altered proteasomal structure and function were observed in both familial and sporadic forms of PD [127]; hence, the UPS is most likely a key factor in the pathogenic process of the disease.

The dysregulation of the UPS and the ALP can also contribute to other neurodegenerative diseases, such as Alzheimer's disease (AD). Numerous genetic and biochemical studies have reported the involvement of the UPS in AD pathogenesis [131, 132]. More specifically, there is evidence linking two hallmark lesions of AD, extracellular plaques, and intracellular neurofibrillary tangles (NFTs), to the UPS. Extracellular plaques are mainly formed by amyloid-beta peptides [133], while NFTs consist of the microtubule-associated protein tau [134]. Amyloid-beta fiber formation has been shown to impair normal proteasomal function [135, 136], which in turn facilitates the accumulation of tau, a proteasomal substrate [131]. Hence, the malfunctioning UPS appears to be both a consequence as well as a contributing factor, with respect to AD.

As mentioned earlier, during the final step of autophagy, the contents of the autolysosome are recycled by autolysosomal proteolysis. Interestingly, in AD, the final proteolysis stage of the autophagy pathway is defective [137, 138], suggesting that ALP failure is a factor in the development of AD-related neurodegeneration. These findings are supported by genetic studies on AD that examined presenilin-1 (PS1) mutations, which are the most common cause of early-onset familial AD (FAD). PS1 is responsible for the activation of lysosomal proteases during autophagy [139, 140]. PS1-null and PS1-FAD fibroblasts and fibroblasts from patients with FAD caused by PS1 mutations show defects in lysosome acidification and autolysosome maturation [141–143].

To summarize, defects in proteasomal or autolysosomal function can lead to inefficient protein clearance and, hence, contribute to the neuronal cell death observed in many different forms of PD and AD. This again points to the key role of the proteases discussed above in several neurodegenerative etiologies.

### 4.1. Interplay between Mitochondrial Dysfunction, PD, and Cancer

Interestingly, there is a strong inverse relationship between neurodegeneration and cancer that is worth noting. The major cause for the progression of neurodegenerative diseases, such as PD, is the death of postmitotic neurons; conversely, in cancer, the central problem is promiscuous cell survival. Studies by several groups have reported an inverse correlation between the risk of developing PD and cancer [144, 145]. Although further study is required to fully understand the inverse connection between the two diseases, the following could be a possible mechanistic explanation for this query.

Neurons are the major cell type affected in PD; however, they are not the only post-mitotic cells. What makes them unique is their high energy demand. Neurons require large amounts of energy to fulfill their normal functions, for which they rely on oxidative phosphorylation [2]. As a result, impairment in the clearance of damaged mitochondrial elements by the quality control systems could be fatal and could lead to neurodegeneration in PD. On the other hand, cancer cells can survive without mitochondria, since they generally exhibit increased glycolysis for their ATP needs [146]. In fact, these cells use mitochondrial clearance as a strategy to enhance their resistance to programmed cell death, which is initiated by mitochondrial proteins [147, 148]. Thus, it is not surprising that mutations that impair mitochondrial clearance would lead to the death of neurons (and PD), but this lack of mitophagy would prevent cancer cells from circumventing apoptosis. In other words, because mitochondrial clearance allows cancer cells to bypass this checkpoint, the mechanism that contributes to PD also promotes the death of malignant cells.

## 5. Overall Perspective

It is clear from the studies outlined above that the UPS has a profound role in many aspects of mitochondrial biology. We have highlighted how mitochondrial-associated DUBs plausibly are novel players in pathways that inevitably lead to proteasomal degradation. Deubiquitinases also have the potential to drive the kinetics of other ubiquitin-mediated pathways such as mitophagy. It is well established that mitophagy is initiated by the accumulation of PINK1 on the OMM. Here, we have discussed how PARL affects PINK1 localization under normal versus stress conditions and, hence, how PARL might add in another layer of regulation to refine the process of mitophagy.

**Figure 6:**
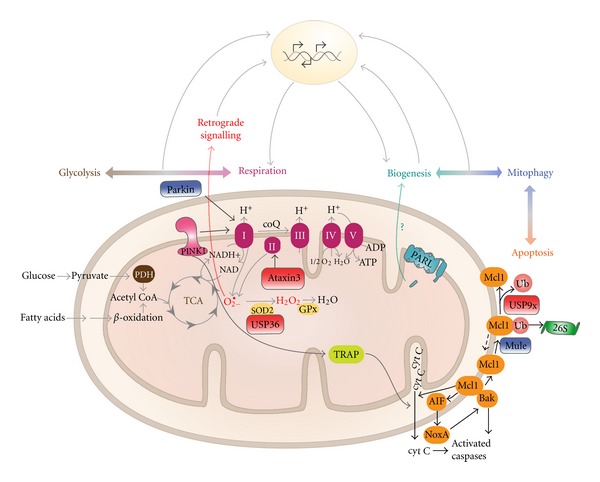
Interdependence of mitochondrial functions. Mitochondria generate ATP energy for the cell with the help of the electron transport chain. ATP fuels many cellular processes including protein degradation by the 26S proteasome. Hence, the process of oxidative phosphorylation (OXPHOS) influences the function of mitochondrial quality control systems. In the reverse direction, components of the quality control pathways can influence OXPHOS. Parkin mutants and defects in PINK1 have been shown to reduce mitochondrial respiration; PINK1 defects impair complex I functionality. In addition, mammalian studies with ataxin-3 mutants showed reduced complex II activity. Mitochondrial dysfunction can trigger apoptosis. Failures within the quality control system, coupled with an increase in ROS, can lead to the release of proapoptotic proteins. For example, if sufficient amounts of Mcl1 are not maintained due to unrestrained proteasomal degradation, Bak can facilitate the release of AIF/cytC and induce apoptosis. Hence, it is important for the quality control mechanisms to function properly in order to prevent unsolicited downstream effects. Overall, the mitochondrial network has to maintain a refined balance between all of these processes and direct effective metabolic outputs depending on the environmental and/or developmental context.

The UPS and mitophagy are two of multiple quality control mechanisms that maintain functional mitochondria. These organelles may have adopted diverse reparative processes so that no one mechanism is overwhelmed at any given time. It is likely that different factors, such as ATP availability, ROS levels, and mitochondrial membrane potential, stimulate certain pathways. Both the UPS and mitophagy require ATP; hence, oxidative phosphorylation has an influence on mitochondrial quality control systems. Conversely, components of mitophagy also influence OXPHOS. Mutations in Parkin as well as defects in PINK1 adversely affect mitochondrial respiration. Furthermore, mutations in ataxin-3 affect complex II activity (Figure 6).

Considering the high interconnectivity of different mitochondrial functions, one of the downstream effects of quality control failure is intrinsic apoptosis. The proper functioning of the mitochondrial UPS is required to maintain sufficient levels of antiapoptotic proteins, such as Mcl1. While, mitophagy is essential for preventing an accumulation of damaged mitochondria in the cell that could elicit cell death. To fully understand the larger implications of mitochondrial quality control systems and mitochondrial dysfunction, we must consider the contributing factors such as energy metabolism and the downstream consequences such as apoptosis (Figure 6). Hence, further studies are required to investigate mitochondrial UPS and mitophagy pathways and to understand how perturbations of these systems relate to overall cellular metabolic states. Because proteolysis is becoming a central theme in regulating all of these integrated pathways and, because proteases are ideal drug targets, there will be intense interest in both academic laboratories and pharmaceutical companies to understand the precise molecular pathways of MAD and mitophagy. New discoveries in these mitochondrial quality control systems and their roles in overall cell integrity will continue to enlighten us on the pathogenesis of neurodegenerative diseases like PD. 

## Figures and Tables

**Table 1:** Functions of known and predicted mitochondrial DUBs.

DUB	Function(s)	Reference(s)
Ataxin-3	Protein quality control; mitophagy; neuroprotective properties	[68, 70–72]
USP9x	Cell differentiation and survival; synaptic function	[63, 65]
USP30	Mitochondrial membrane dynamics	[59]
USP36	Nucleolar structure and function; oxidative stress	[61, 62]

JOSD1	Unknown	
USP2	NF-kappaB signalling; AIF-mediated cell death	[73, 74]
USP16	Histone deubiquitination; gene expression	[75]
USP29	Oxidative stress	[76]
USP44	Cell cycle regulation	[50]
USP50	Cell cycle regulation	[77]
